# Reasons for using electronic cigarettes among young adults aged 18 – 30: a systematic review

**DOI:** 10.17179/excli2024-8085

**Published:** 2025-01-31

**Authors:** Shérazade Kinouani, Faustine Roux, Bastien Questel, Maëlys Abraham, Christophe Tzourio

**Affiliations:** 1 University of Bordeaux, Inserm, Bordeaux Population Health Research Center, team HEALTHY, UMR 1219, Bordeaux, France; 2 Department of General Practice, University of Bordeaux, Bordeaux, France; 3 48 avenue de Labarde, Bordeaux, France

**Keywords:** young adults, electronic nicotine delivery systems, vaping

## Abstract

The use of psychoactive products by young adults is usually described as part of their exploratory identity development. This behavior is facilitated by social and structural contexts where these substances are perceived as legal and easily accessible. While the motivations for initiating and continuing the use of tobacco and alcohol are well-documented, the same cannot be said for e-cigarettes. The primary objective of this systematic review was to describe the reasons for initiation and continuation of e-cigarette use among adults aged 18 to 30. A secondary objective was to categorize these reasons into intrinsic (i.e., personal motivations) and extrinsic factors (i.e., sociocultural or structural influences). Searches were conducted in MEDLINE, Scopus, SocINDEX full text, PsycArticles, PsycInfo, Psychology and Behavioral Sciences Collection, Cochrane Library and gray literature. Studies involving humans, published in English or French up to June 2024 were eligible for inclusion. After removing 594 duplicates, 1,123 articles were screened by title and abstract, with 37 articles published between 2015 and 2024 ultimately included in the review. These comprised 21 cross-sectional studies, eight qualitative studies, six cross-sectional analyses of cohort data, one cohort study and one mixed methods study. The appeal of e-liquid flavors emerged as one of the most frequently reported extrinsic factors driving both initiation and continuation of e-cigarette use. Other reasons varied across intrinsic and extrinsic domains: smoking cessation was a commonly cited intrinsic motivation, often reported alongside other factors. Structural extrinsic factors such as regulatory policies appeared to be less recognized by young adults, suggesting a gap in awareness or compliance to such regulations. These findings indicate the need for further research to better understand young adults' perceptions of and interactions with regulatory measures concerning e-cigarette and tobacco use.

## Abbreviations

95 % CI: 95 % Confidence interval

COREQ: COnsolidated criteria for REporting Qualitative research

HIV: Human Immunodeficiency Virus

MMAT: Mixed Methods Appraisal Tool

MMARS: Mixed Methods Article Reporting Standards

OECD: Organisation for Economic Co-operation and Development

PAP: Psychoactive Products

STROBE: Strengthening the Reporting of Observational Studies in Epidemiology

UK: United Kingdom

## Introduction

The relationship between young adults aged 18-30 and psychoactive products (PAP) has been studied extensively by psychologists and sociologists. Jeffrey Jensen Arnett theorized that risk-taking behavior during emerging adulthood is a fundamental component of identity exploration. The PAP consumption reflects young adults' desire to accumulate diverse experiences before transitioning into the roles and responsibilities of adulthood (Arnett, 2000[3], 2005[6]; Arnett et al., 2014[7]). The friendships formed or deepened during this period exert limited influence in terms of social control, further enabling such behavior. Having friends who smoke tobacco has been identified as a significant predictor of tobacco initiation (Steinmetz-Wood et al., 2018[75]). The first time trying an e-cigarette is almost always done in the presence of friends or a family member who already uses one (McKeganey et al., 2018[54]); having friends or living with someone who vapes is associated with the risk of later initiation of e-cigarette use (Urman et al., 2019[82]). Another explanation for the relationship between young adults and PAP is found in the Sociology of deviance (Becker, 1963[10]; Peretti-Watel et al., 2007[65][64][66]). Behavior is classified as deviant when it causes social problems for the social group that sets the norms in a given time and space. In his research, Howard Becker examined the experiences of American cannabis smokers and described their "moral career" as a diachronic trajectory of cannabis use that is constructed in both objective and subjective ways (Becker, 1963[10]). From an objective point of view, a user's career consists of successive stages: experimentation, occasional use, and then regular use. Going beyond simple experimentation requires repeated and progressive learning about the use and effects of the product. As Peretti-Watel explained, "*experimentation would be motivated primarily by curiosity, with the desired effects becoming a motivation only when the individual has learned to perceive them and develop a taste for them*” (Peretti-Watel et al., 2007[65]). Experimentation requires both that the product be accessible and socially acceptable (extrinsic factors) and that the subject be willing to try it (intrinsic factors). Regular or occasional use in addition requires that the subject be inclined to learn how to use it (intrinsic factors). Finally, from a subjective point of view, consumers neutralize social stereotypes and re-adjust their own opinions at each stage of the objective moral career in order to continue to see themselves as non-deviant. The Sociology of deviance emphasizes the importance of distinguishing between the reasons for experimenting with a PAP and the reasons for continuing to use it. To our knowledge, this theory has been little explored in the context of e-cigarette use.

A projection based on data from the National Health Interview Survey indicates that the prevalence of smoking in the United States has declined at an accelerated rate over the past decade, in line with the observed increase in the use of e-cigarettes, also known as vaping. The prevalence of smoking observed following the introduction of e-cigarettes in the United States in 2007 was lower than expected based on trends between 1990 and 2006, particularly among individuals aged 18-34 (Foxon et al., 2024[24]). Regarding e-cigarette use among young adults, the prevalence of current e-cigarette use (defined as occasional or daily use at the time of the survey) among Americans aged 18-24 in 2021 was estimated to be 11 % (Cornelius et al., 2023[21]; Kramarow and Elgaddal, 2023[48]). Exclusive use of e-cigarettes was more common than exclusive use of tobacco cigarettes or concurrent use of tobacco cigarettes and e-cigarettes (dual use). In 2017, the prevalence of regular e-cigarette use (defined as weekly or daily use) was estimated at 1.8 % in a representative sample of Europeans aged 15 and older. This represents an increase in use of 21.2 % between 2014 and 2017 (Laverty et al., 2018[49]). Compared with those aged 55 or older, those aged 15-24 were more likely to have tried e-cigarettes in 2017, but less likely to be regular users (Laverty et al., 2018[49]). In 2019, the prevalence of current use (defined as occasional or daily use at the time of the survey) was 5.1 % among Europeans aged 15-24 (OECD/European Union, 2022[59]). 

The factors driving young adults to use e-cigarettes (also referred to as electronic cigarettes, electronic nicotine delivery systems, or vaping devices) appear to be less well-documented compared to those associated with tobacco or alcohol use. However, similar to tobacco and alcohol, the sale and use of e-cigarettes by young adults is permitted in most developed countries. This permissive structural context may have contributed to their growing interest in vaping products. 

To better understand such behavior, we conducted a literature review to identify and distinguish between the reasons for initiating e-cigarette use and those for its continued use, conceptualizing these as two discrete stages in the trajectory of e-cigarette consumption. We hypothesized that intrinsic factors (i.e., personal motivations) and extrinsic factors (i.e., sociocultural or structural influences) would complement each other in shaping vapers' trajectories. 

Thus, the primary objective of this systematic review was to describe the reasons for initiating and maintaining e-cigarette use among individuals aged 18-30. The secondary aim was to categorize these reasons into intrinsic and extrinsic factors to provide a comprehensive framework for understanding the dynamics of vaping behaviors within this age group.

## Materials and Methods

This systematic review was conducted according to the Preferred Reporting Items for Systematic Reviews and Meta-analyses (PRISMA) guidelines (Page et al., 2021[62]). The protocol details were registered in Prospero (number CRD42024553490).

### Data sources and search strategy

We searched the following electronic databases for articles published in English or French up to June 2, 2024: MEDLINE, Scopus, SocINDEX with full text, PsycArticles, PsycInfo, Psychology and Behavioral Sciences Collection, Cochrane Library. For gray literature, we used an international search engine BASE (https://www.base-search.net/). The list of search equations is shown in Table 1(Tab. 1). Two hundred and six additional records were identified through analysis of the references of the included articles and through consultation with members of the research team.

### Article screening

#### Population eligibility criteria

We focused this review on adults between the ages of 18 and 30. Participants had to have used e-cigarettes at least once. If multiple age groups were described in the articles, only reasons for using e-cigarettes related to adults aged 18 to 30 were selected. Articles focusing on specific comorbidity populations (such as those with HIV, mental illness, chronic disease, etc.) or lacking sufficient information to estimate the age of participants were excluded. Articles with insufficient information to estimate the number of young adult users to be considered were also excluded.

#### Article eligibility criteria

All study designs were included. We defined reasons for use as reasons reported by e-cigarette users: i) to initiate or try e-cigarettes for the first time (reasons for initiation); ii) to continue using after initiation, regardless of frequency or duration of use (reasons for continuation). Articles that reported only intentions to use among non-users were excluded from the analysis. Articles that solely addressed awareness, representations or perceptions of e-cigarette use without exploring reasons for use were excluded from the review. Articles were also excluded if it was not clear whether the reasons explored were related to initiation or continuation of e-cigarette use. 

#### Article selection

First, two trained students (FR and BQ) selected articles and independently evaluated them on title and abstract. They tried to resolve their disagreements by consensus. A third reviewer (SK) read the 11 articles where students disagreed. She included 10 and excluded one. Then FR and BQ read the full text of the selected articles independently. Their reviewers' agreement was assessed using Cohen's unweighted kappa coefficient, which was estimated to be 0.488 (95 % CI: 0.330-0.645). This indicated moderate agreement. They were able to reach full agreement after discussing their differences without the need for a third reviewer. These two steps were performed using Rayyan software (Ouzzani et al., 2016[60]).

### Data extraction, synthesis and quality assessment

Each student extracted information from half of the documents selected in the previous phase. Each extraction was fully verified by SK. The following information was extracted: location, study design, data collection strategy, participant characteristics, definitions and reasons for using e-cigarettes.

Each student was responsible for assessing the quality of half of the studies. SK conducted a similar assessment independently for all articles. They all used the Mixed Methods Appraisal Tool or MMAT (Pace et al., 2012[61]; Souto et al., 2015[74]; Hong et al., 2018[36], 2019[37]). SK compared her MMAT score with that of the student's to reach a consensus for each study. The MMAT score was decided as follows: 0-2: poor quality; 3-4: fair quality; 5: good quality. SK also assessed the quality of reporting in all articles using the appropriate assessment tools: STROBE for cross-sectional or cohort studies (von Elm et al., 2007[84]), COREQ for qualitative studies (Tong et al., 2007[80]), MMARS for mixed methods studies (Levitt et al., 2018[51]). The quality of reporting was classified as poor, fair, or good. Finally, SK assessed a combination of the quality of reporting in the article and the quality of the study, according to the rules presented in Supplementary information (Table S1).

SK conducted the synthesis on reasons for e-cigarette use, first dividing them into reasons for initiation and reasons for continued use. She then distinguished intrinsic reasons from extrinsic reasons. During her analysis, it appeared that reasons related to vaping product features were a separate category of extrinsic factors. She decided to individualize them.

All studies that were retained at the conclusion of the full-text reading selection were included in the main analysis, irrespective of their global quality assessment. A robustness analysis was conducted by removing studies of low quality or with declared conflicts of interest.

## Results

### Description of studies

**Figure 1:**
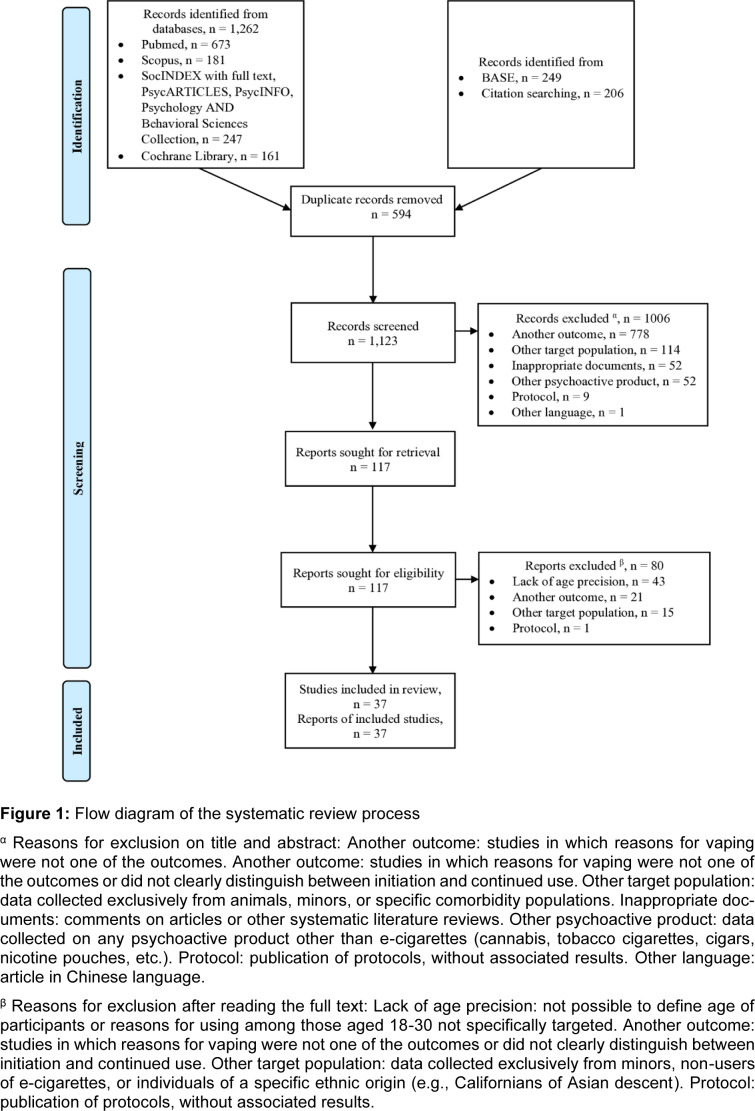
Flow diagram of the systematic review process ^α^ Reasons for exclusion on title and abstract: Another outcome: studies in which reasons for vaping were not one of the outcomes. Another outcome: studies in which reasons for vaping were not one of the outcomes or did not clearly distinguish between initiation and continued use. Other target population: data collected exclusively from animals, minors, or specific comorbidity populations. Inappropriate documents: comments on articles or other systematic literature reviews. Other psychoactive product: data collected on any psychoactive product other than e-cigarettes (cannabis, tobacco cigarettes, cigars, nicotine pouches, etc.). Protocol: publication of protocols, without associated results. Other language: article in Chinese language. ^β^ Reasons for exclusion after reading the full text: Lack of age precision: not possible to define age of participants or reasons for using among those aged 18-30 not specifically targeted. Another outcome: studies in which reasons for vaping were not one of the outcomes or did not clearly distinguish between initiation and continued use. Other target population: data collected exclusively from minors, non-users of e-cigarettes, or individuals of a specific ethnic origin (e.g., Californians of Asian descent). Protocol: publication of protocols, without associated results.

After removing duplicates, 1123 documents were identified. Screening based on title and abstract allowed 117 documents to be retained. After reading the full text, 37 were finally retained for the systematic review, describing 37 different studies. The flow diagram is shown in Figure 1(Fig. 1). These included 35 English-language articles, one English-language study report (Meng and Ponce, 2020[55]), and one French-language study report (Chok et al., 2023[19]) (see Table 2(Tab. 2) for all studies used; References In Table 2: Awan, 2016[8]; Bunch et al., 2018[14]; Buu et al., 2020[15]; Case et al., 2020[16]; Cheney et al., 2016[17]; Chok et al., 2023[19]; Cooper et al., 2016[20]; Davis et al., 2024[22]; Dunlop et al., 2016[23]; Freeman et al., 2023[25]; Hoek et al., 2017[34]; Hong et al., 2019[35]; Ickes et al., 2020[39]; Jongenelis et al., 2019[40]; Kava et al., 2021[41]; Kechter et al., 2022[42]; Khouja et al., 2020[43]; Kinouani et al., 2017[46], 2024[45]; Lotrean, 2015[52]; Martinasek et al., 2018[53]; Meng and Ponce, 2020[55]; Newcombe et al., 2021[56]; Obisesan et al., 2023[58]; Patel et al., 2016[63]; Pettigrew et al., 2023[67][68]; Pinho-Gomes et al., 2023[69]; Robertson et al., 2022[70]; Roh et al., 2024[71]; Rostron et al., 2020[72]; Stone et al., 2023[76]; Tamulevicius et al., 2020[78]; Thoonen and Jongenelis, 2024[79]; Tran et al., 2024[81]; Vu et al., 2018[85], 2019[86]). As shown in Table 2(Tab. 2), the articles were published between 2015 and the first five months of 2024: one in 2015, five in 2016, two in 2017, three in 2018, three in 2019, seven in 2020, two in 2021, two in 2022, seven in 2023, and five in 2024. Twenty-one studies were conducted in the United States, five in Australia, two in New Zealand, two in the United Kingdom (UK), two in France, one in China, one in Switzerland, one in Romania, one in Spain, and one in Saudi Arabia. One study was conducted simultaneously in three countries: United States, Germany, and South Africa. Studies included 21 cross-sectional studies, 6 cross-sectional analyses of cohort data, one cohort with 12 months of follow-up, 8 qualitative studies using thematic analysis, and one study using mixed methods (Table 2(Tab. 2)). Some studies were conducted in specific subgroups based on student status, smoking status, or type of electronic device used (Tables 3(Tab. 3) and 4(Tab. 4)). There were 14 studies carried out among college students, one among young adults who had not completed higher education (Cheney et al., 2016[17]), one exclusively among young adults who had never smoked tobacco before vaping (Tran et al., 2024[81]), one among subjects who had never "smoked cigarettes regularly" (Robertson et al., 2022[70]), one among former or current tobacco smokers (Dunlop et al., 2016[23]). There were also five studies among pod users (Case et al., 2020[16]; Ickes et al., 2020[39]; Kava et al., 2021[41]; Newcombe et al., 2021[56]; Obisesan et al., 2023[58]), one study among users of disposable e-cigarettes called puffs (Chok et al., 2023[19]), and one study focused on the use of synthetic nicotine (tobacco-free e-cigarettes) (Davis et al., 2024[22]).

As shown in Supplementary information (Table S2), e-cigarette use was defined in different ways, whether as initiation or continuation. Studies often referred to the first try or experience (even if it was only 1 or 2 puffs) as initiated use. Continued use ranged from current use at the time of the survey (regardless of frequency) to use over at least 6 months (with the participant bringing their most recent device to the interview). The definition used in the quantitative studies was not always clear: three studies concerned current use without ever defining it or giving more details (Dunlop et al., 2016[23]; Bunch et al., 2018[14]; Khouja et al., 2020[43]). However, most studies referred to use in the past 30 days or in the past month.

### Reasons for initiating use

Eighteen studies reported reasons for initiation of cigarette use among young adults (Table 3(Tab. 3); References in Table 3: Awan, 2016[8]; Cheney et al., 2016[17]; Davis et al., 2024[22]; Ickes et al., 2020[39]; Kava et al., 2021[41]; Kechter et al., 2022[42]; Kinouani et al., 2017[46], 2024[45]; Lotrean, 2015[52]; Martinasek et al., 2018[53]; Obisesan et al., 2023[58]; Robertson et al., 2022[70]; Roh et al., 2024[71]; Tamulevicius et al., 2020[78]; Thoonen and Jongenelis, 2024[79]; Tran et al., 2024[81]; Vu et al., 2018[85], 2019[86]). According to the 11 quantitative studies and the quantitative phase of the mixed methods study, reasons for initiating e-cigarette use included: out of curiosity (Lotrean, 2015[52]; Awan, 2016[8]; Kinouani et al., 2017[46], 2024[45]; Ickes et al., 2020[39]; Obisesan et al., 2023[58]; Davis et al., 2024[22]; Roh et al., 2024[71]; Thoonen and Jongenelis, 2024[79]) or the desire to try new things (Vu et al., 2019[86]; Tamulevicius et al., 2020[78]); appeal of flavors (Kinouani et al., 2017[46], 2024[45]; Martinasek et al., 2018[53]; Vu et al., 2019[86]; Ickes et al., 2020[39]; Tamulevicius et al., 2020[78]; Obisesan et al., 2023[58]; Davis et al., 2024[22]; Thoonen and Jongenelis, 2024[79]) in terms of variety, accessibility, and availability; perception of vaping as less harmful to health than smoking (Lotrean, 2015[52]; Awan, 2016[8]; Kinouani et al., 2017[46], 2024[45]; Vu et al., 2019[86]; Obisesan et al., 2023[58]; Davis et al., 2024[22]; Roh et al., 2024[71]; Thoonen and Jongenelis, 2024[79]); quitting smoking (Lotrean, 2015[52]; Awan, 2016[8]; Kinouani et al., 2017[46], 2024[45]; Martinasek et al., 2018[53]; Vu et al., 2019[86]; Tamulevicius et al., 2020[78]; Davis et al., 2024[22]; Thoonen and Jongenelis, 2024[79]) or reducing smoking (Kinouani et al., 2017[46], 2024[45]; Obisesan et al., 2023[58]; Thoonen and Jongenelis, 2024[79]); using vaping to avoid the inconveniences of smoking such as bans on smoking in certain places (Kinouani et al., 2017[46], 2024[45]; Obisesan et al., 2023[58]; Thoonen and Jongenelis, 2024[79]), to avoid the bad smell of smoking (Vu et al., 2019[86]; Ickes et al., 2020[39]; Obisesan et al., 2023[58]; Davis et al., 2024[22]; Thoonen and Jongenelis, 2024[79]), or exposure to second-hand smoke (Obisesan et al., 2023[58]; Kinouani et al., 2024[45]). Having people around you who used tobacco or e-cigarettes also facilitated initiation: having friends (Lotrean, 2015[52]; Awan, 2016[8]; Vu et al., 2019[86]; Ickes et al., 2020[39]; Tamulevicius et al., 2020[78]; Roh et al., 2024[71]) or family (Roh et al., 2024[71]) who had already vaped, having friends who suggested trying for the first time by sharing their e-cigarette (Kinouani et al., 2017[46], 2024[45]; Obisesan et al., 2023[58]). Perceiving vaping as cheaper than smoking (Kinouani et al., 2017[46], 2024[45]; Vu et al., 2019[86]; Davis et al., 2024[22]; Thoonen and Jongenelis, 2024[79]) or as a cool practice (Martinasek et al., 2018[53]; Tamulevicius et al., 2020[78]; Davis et al., 2024[22]; Thoonen and Jongenelis, 2024[79]) were other reported reasons for initiation, as was the fun aspect of vaping tricks (Vu et al., 2019[86]; Kinouani et al., 2024[45]; Roh et al., 2024[71]). Less commonly reported reasons in quantitative studies included: trying vaping to manage stress or anxiety (Martinasek et al., 2018[53]; Davis et al., 2024[22]; Roh et al., 2024[71]); to manage tobacco cravings (Obisesan et al., 2023[58]; Davis et al., 2024[22]), to control weight or appetite (Martinasek et al., 2018[53]; Tamulevicius et al., 2020[78]), to get an energy boost (Davis et al., 2024[22]), to avoid smoking relapse (Thoonen and Jongenelis, 2024[79]), for ease of use (Ickes et al., 2020[39]), for availability of e-cigarettes (Davis et al., 2024[22]; Roh et al., 2024[71]), for discretion of use (Kinouani et al., 2024[45]; Roh et al., 2024[71]), because of social acceptability (Davis et al., 2024[22]; Thoonen and Jongenelis, 2024[79]), perceiving vaping as similar to smoking (Obisesan et al., 2023[58]; Kinouani et al., 2024[45]), perceiving it as a pleasant and enjoyable practice (Davis et al., 2024[22]), combining vaping with alcohol or cannabis use (Davis et al., 2024[22]), appeal of advertising (Obisesan et al., 2023[58]), seeing a celebrity (on TV or online) who vape (Roh et al., 2024[71]) or recommendation of use by a healthcare professional (Vu et al., 2019[86]). Some of these studies suggested that curiosity was the main reason for experimenting with e-cigarettes, regardless of smoking status (Lotrean, 2015[52]; Awan, 2016[8]; Kinouani et al., 2017[46]; Thoonen and Jongenelis, 2024[79]).

According to the six qualitative studies and the mixed methods study, flavors had an attractive potential (Cheney et al., 2016[17]; Vu et al., 2018[85]), especially because of their variety (Kechter et al., 2022[42]). They reported that e-cigarettes were perceived as a less harmful alternative to smoking (Vu et al., 2018[85]; Kechter et al., 2022[42]; Kinouani et al., 2024[45]). Perceived social acceptability (Kechter et al., 2022[42]) was also a factor promoting initiation. A family member may have encouraged the initiation of vaping either following their own vaping experience (Kinouani et al., 2024[45]), or by insisting that smokers quit tobacco (Cheney et al., 2016[17]). Experimentation may also have been encouraged by peers (Kechter et al., 2022[42]). The first try often took place with vaping friends (Cheney et al., 2016[17]; Vu et al., 2018[85]) who shared their electronic devices for the occasion (Cheney et al., 2016[17]; Kava et al., 2021[41]; Robertson et al., 2022[70]; Kinouani et al., 2024[45]). Curiosity led to trying an e-cigarette when in contact with these vaping friends (Kechter et al., 2022[42]; Tran et al., 2024[81]), as did the fun and playful aspect of this shared experience (Robertson et al., 2022[70]). E-cigarettes were described as facilitating social interactions (Kava et al., 2021[41]; Kinouani et al., 2024[45]) and integration (Tran et al., 2024[81]) among peers. Some qualitative studies also described initiating e-cigarettes as a means to circumvent smoking bans in certain places (Vu et al., 2018[85]), to cope with stress (Tran et al., 2024[81]), boredom (Tran et al., 2024[81]), to suppress appetite (Tran et al., 2024[81]), to quit smoking (Vu et al., 2018[85]; Kinouani et al., 2024[45]) or reduce smoking (Kinouani et al., 2024[45]). E-cigarettes were described by some young adults as easier and more enjoyable to use than traditional nicotine replacement therapies (Kinouani et al., 2024[45]).

The most common extrinsic factors for e-cigarette experimentation reported in the articles were the appeal of e-liquid flavors and the opportunity to try it with close vapers (family, friends, and peers). Curiosity, the perception that vaping is less harmful than smoking, and quitting smoking were the most reported intrinsic factors for experimentation (Supplementary information, Table S3).

### Reasons for continuing use

Twenty-eight studies reported reasons for continued vaping among young adults (Table 4(Tab. 4); References in Table 4: Bunch et al., 2018[14]; Buu et al., 2020[15]; Case et al., 2020[16]; Cheney et al., 2016[17]; Chok et al., 2023[19]; Cooper et al., 2016[20]; Dunlop et al., 2016[23]; Freeman et al., 2023[25]; Hoek et al., 2017[34]; Hong et al., 2019[35]; Ickes et al., 2020[39]; Jongenelis et al., 2019[40]; Kava et al., 2021[41]; Kechter et al., 2022[42]; Khouja et al., 2020[43]; Kinouani et al., 2024[45]; Meng and Ponce, 2020[55]; Newcombe et al., 2021[56]; Patel et al., 2016[63]; Pettigrew et al., 2023[67][68]; Pinho-Gomes et al., 2023[69]; Robertson et al., 2022[70]; Roh et al., 2024[71]; Rostron et al., 2020[72]; Stone et al., 2023[76]; Thoonen and Jongenelis, 2024[79]; Vu et al., 2018[85]). According to the 20 qualitative studies and the mixed methods study, the most common reasons for continued e-cigarette use in the studies were: quitting smoking (Dunlop et al., 2016[23]; Patel et al., 2016[63]; Bunch et al., 2018[14]; Hong et al., 2019[35]; Jongenelis et al., 2019[40]; Buu et al., 2020[15]; Ickes et al., 2020[39]; Khouja et al., 2020[43]; Meng and Ponce, 2020[55]; Chok et al., 2023[19]; Freeman et al., 2023[25]; Pettigrew et al., 2023[67][68]; Pinho-Gomes et al., 2023[69]; Stone et al., 2023[76]; Kinouani et al., 2024[45]; Thoonen and Jongenelis, 2024[79]); appeal of flavors (Patel et al., 2016[63]; Hong et al., 2019[35]; Buu et al., 2020[15]; Ickes et al., 2020[39]; Rostron et al., 2020[72]; Newcombe et al., 2021[56]; Chok et al., 2023[19]; Pettigrew et al., 2023[67][68]; Pinho-Gomes et al., 2023[69]; Stone et al., 2023[76]; Kinouani et al., 2024[45]; Roh et al., 2024[71]; Thoonen and Jongenelis, 2024[79]); less harm perceived for self or others in vaping compared to smoking (Dunlop et al., 2016[23]; Patel et al., 2016[63]; Hong et al., 2019[35]; Buu et al., 2020[15]; Ickes et al., 2020[39]; Chok et al., 2023[19]; Freeman et al., 2023[25]; Pinho-Gomes et al., 2023[69]; Stone et al., 2023[76]; Kinouani et al., 2024[45]; Roh et al., 2024[71]; Thoonen and Jongenelis, 2024[79]); lower cost compared with smoking (Dunlop et al., 2016[23]; Patel et al., 2016[63]; Hong et al., 2019[35]; Buu et al., 2020[15]; Ickes et al., 2020[39]; Chok et al., 2023[19]; Freeman et al., 2023[25]; Pinho-Gomes et al., 2023[69]; Stone et al., 2023[76]; Kinouani et al., 2024[45]; Thoonen and Jongenelis, 2024[79]); reduction in the amount of tobacco consumed (Dunlop et al., 2016[23]; Patel et al., 2016[63]; Bunch et al., 2018[14]; Khouja et al., 2020[43]; Meng and Ponce 2020[55]; Chok et al., 2023[19]; Freeman et al., 2023[25]; Pettigrew et al., 2023[67][68]; Pinho-Gomes et al., 2023[69]; Kinouani et al., 2024[45]; Thoonen and Jongenelis, 2024[79]); the possibility of vaping in places where smoking is prohibited (Dunlop et al., 2016[23]; Patel et al., 2016[63]; Bunch et al., 2018[14]; Hong et al., 2019[35]; Buu et al., 2020[15]; Ickes et al., 2020[39]; Freeman et al., 2023[25]; Pinho-Gomes et al., 2023[69]; Kinouani et al., 2024[45]; Thoonen and Jongenelis, 2024[79]); seeking the psychotropic effects of nicotine to cope with stress and anxiety or to concentrate (Case et al., 2020[16]; Ickes et al., 2020[39]; Newcombe et al., 2021[56]; Pinho-Gomes et al., 2023[69]; Stone et al., 2023[76]; Kinouani et al., 2024[45]; Roh et al., 2024[71]); because of important close people (family, but especially friends) use them (Hong et al., 2019[35]; Buu et al., 2020[15]; Ickes et al., 2020[39]; Khouja et al., 2020[43]; Chok et al., 2023[19]; Pettigrew et al., 2023[67][68]; Pinho-Gomes et al., 2023[69]; Roh et al., 2024[71]); out of curiosity (Patel et al., 2016[63]; Ickes et al., 2020[39]; Khouja et al., 2020[43]; Meng and Ponce, 2020[55]; Chok et al., 2023[19]; Freeman et al., 2023[25]; Pettigrew et al., 2023[67][68]; Pinho-Gomes et al., 2023[69]; Thoonen and Jongenelis, 2024[79]); the taste of vaping was perceived as more pleasant than that of smoking (Dunlop et al., 2016[23]; Hong et al., 2019[35]; Freeman et al., 2023[25]; Pettigrew et al., 2023[67][68]; Pinho-Gomes et al., 2023[69]; Thoonen and Jongenelis, 2024[79]) and because it is a fun and enjoyable practice (Dunlop et al., 2016[23]; Hong et al., 2019[35]; Freeman et al., 2023[25]; Pettigrew et al., 2023[67][68]; Pinho-Gomes et al., 2023[69]; Thoonen and Jongenelis, 2024[79]). To a lesser extent, the following reasons were reported: to appear cool (Jongenelis et al., 2019[40]; Case et al., 2020[16]; Ickes et al., 2020[39]; Chok et al., 2023[19]; Kinouani et al., 2024[45]; Thoonen and Jongenelis, 2024[79]); the better smell it produces (Hong et al., 2019[35]; Chok et al., 2023[19]) or the absence of (bad) smell compared to smoking (Patel et al., 2016[63]; Buu et al., 2020[15]; Ickes et al., 2020[39]; Pinho-Gomes et al., 2023[69]; Stone et al., 2023[76]); the practicality of use (Ickes et al., 2020[39]; Newcombe et al., 2021[56]; Chok et al., 2023[19]; Kinouani et al., 2024[45]). Vaping facilitated social interactions and integration (Buu et al., 2020[15]; Case et al., 2020[16]; Meng and Ponce, 2020[55]; Newcombe et al., 2021[56]; Kinouani et al., 2024[45]); it was described as more acceptable than smoking (Hong et al., 2019[35]; Pinho-Gomes et al., 2023[69]; Stone et al., 2023[76]; Kinouani et al., 2024[45]; Thoonen and Jongenelis, 2024[79]), especially by non-smokers (Patel et al., 2016[63]; Hong et al., 2019[35]; Buu et al., 2020[15]). Young adults have also described the following reasons: the similarities between vaping and smoking (Patel et al., 2016[63]; Hong et al., 2019[35]; Buu et al., 2020[15]; Kinouani et al., 2024[45]); the appeal of advertising (Hong et al., 2019[35]; Buu et al., 2020[15]; Stone et al., 2023[76]), especially on social networks (Ickes et al., 2020[39]); seeing people vaping in public and in the media (Hong et al., 2019[35]; Buu et al., 2020[15]; Roh et al., 2024[71]); discretion of use (Chok et al., 2023[19]; Kinouani et al., 2024[45]; Roh et al., 2024[71]), especially because e-cigarette use could be hidden from adults (Ickes et al., 2020[39]); for appetite or weight control (Case et al., 2020[16]; Newcombe et al., 2021[56]; Kinouani et al., 2024[45]), for an energy boost (Case et al., 2020[16]; Ickes et al., 2020[39]; Newcombe et al., 2021[56]) or just for a nicotine hit (Case et al., 2020[16]; Kinouani et al., 2024[45]; Roh et al., 2024[71]); for the possibility of personalizing use (Ickes et al., 2020[39]; Chok et al., 2023[19]; Kinouani et al., 2024[45]); for preventing smoking relapse (Pinho-Gomes et al., 2023[69]; Thoonen and Jongenelis, 2024[79]) or smoking initiation (Kinouani et al., 2024[45]); for ease of purchase (Ickes et al., 2020[39]; Roh et al., 2024[71]), transportation (Ickes et al., 2020[39]), or refilling (Ickes et al., 2020[39]); as a way to reduce the risks associated with tobacco (Case et al., 2020[16]), such as consuming less nicotine (Chok et al., 2023[19]); to manage cravings (Patel et al., 2016[63]; Khouja et al., 2020[43]; Kinouani et al., 2024[45]); to avoid going out to smoke (Kinouani et al., 2024[45]); to take a few puffs without having to finish a cigarette (Kinouani et al., 2024[45]); the lower perceived addictiveness compared to tobacco (Kinouani et al., 2024[45]); the opportunity to do vaping tricks (Roh et al., 2024[71]); to appear more mature (Case et al., 2020[16]); to manage symptoms of a chronic disease such as headaches (Newcombe et al., 2021[56]); because they had received a discount coupon for their purchase (Ickes et al., 2020[39]). Young adults also reported frequently using e-cigarettes while drinking alcohol (Newcombe et al., 2021[56]).

Three quantitative studies have examined the reasons for continued use of e-cigarettes based on smoking status. The results of these studies are inconsistent. In one study, the taste of e-cigarettes and the appeal of flavors were the main reasons reported by both smokers and nonsmokers (Thoonen and Jongenelis, 2024[79]). In another study, viewing vaping as fun or pleasurable was the main reason reported regardless of smoking status (Jongenelis et al., 2019[40]). Finally, in the third study, quitting and then reducing smoking were the top two reasons for continued use among 23-year-old young adults who had smoked tobacco before vaping (Khouja et al., 2020[43]).

According to the seven qualitative studies and the qualitative component of the mixed methods study, the reasons for continuing to use e-cigarettes varied. They could be used to quit smoking (Cheney et al., 2016[17]; Vu et al., 2018[85]; Kinouani et al., 2024[45]) or to reduce tobacco smoking (Cheney et al., 2016[17]; Kinouani et al., 2024[45]). Young adult tobacco smokers also used e-cigarettes to manage signs of a tobacco use disorder or to reduce physical complications associated with chronic use: to prevent relapse after quitting smoking (Cheney et al., 2016[17]; Kinouani et al., 2024[45]), to manage withdrawal symptoms (Kinouani et al., 2024[45]) or to preserve their lungs or voice (Cheney et al., 2016[17]; Hoek et al., 2017[34]; Kava et al., 2021[41]; Robertson et al., 2022[70]; Kinouani et al., 2024[45]). Some participants who perceived advantages of vaping over smoking favored continued use of e-cigarettes: perceived less harm (Vu et al., 2018[85]; Kava et al., 2021[41]; Kinouani et al., 2024[45]); lower cost than smoking (Hoek et al., 2017[34]; Robertson et al., 2022[70]; Kinouani et al., 2024[45]); vaping in places where smoking is prohibited (Vu et al., 2018[85]; Kechter et al., 2022[42]; Kinouani et al., 2024[45]); absence of odor (Kinouani et al., 2024[45]); less disruptive than tobacco to those around them (Kinouani et al., 2024[45]). Some vaped for the effects of nicotine: to cope with stress or improve concentration (Cheney et al., 2016[17]; Cooper et al., 2016[20]; Vu et al., 2018[85]; Kava et al., 2021[41]; Robertson et al., 2022[70]; Kinouani et al., 2024[45]); to control appetite (Robertson et al., 2022[70]; Kinouani et al., 2024[45]) or negative emotions (Robertson et al., 2022[70]). While some young adult smokers used e-cigarettes as an alternative to cigarettes, trying to maintain smoking rituals or rhythms (Hoek et al., 2017[34]; Kinouani et al., 2024[45]), others used vaping as a way to move away from smoking by adopting a new practice (Hoek et al., 2017[34]; Kinouani et al., 2024[45]); they may use their electronic devices as a marker of identity (Robertson et al., 2022[70]; Kinouani et al., 2024[45]) or develop a vaping expertise that they share (Hoek et al., 2017[34]; Kinouani et al., 2024[45]). Young adults reported vaping for fun (Cheney et al., 2016[17]) or to pass the time/relieve boredom (Cheney et al., 2016; Robertson et al., 2022[70]). The technological features of electronic devices (Cooper et al., 2016[20]) and the possibility of vaping tricks (Cheney et al., 2016[17]; Cooper et al., 2016[20]; Kava et al., 2021[41]) contributed to vaping for fun. Vaping tricks had also allowed some of them to gain visibility and recognition as artistic performers (Robertson et al., 2022[70]). Continuing to vape was also justified by its usefulness in starting conversations with strangers (Cheney et al., 2016[17]), and maintaining social connections with smoking friends (Hoek et al., 2017[34]; Kinouani et al., 2024[45]). Vaping facilitated social interactions (Kava et al., 2021[41]; Robertson et al., 2022[70]; Kinouani et al., 2024[45]); it also allowed integration within a group of smoking peers (Robertson et al., 2022[70]). Some reasons for continuing to vape were related to the characteristics of e-cigarettes and e-liquids (and not related to tobacco smoking): the appeal of the variety of flavors (Cheney et al., 2016[17]; Cooper et al., 2016[20]; Vu et al., 2018[85]; Kava et al., 2021[41]; Robertson et al., 2022[70]; Kinouani et al., 2024[45]); the possibility to customize e-cigarette use (Cheney et al., 2016[17]; Cooper et al., 2016[20]; Kinouani et al., 2024[45]), especially through “do-it-yourself” (or DIY) (Kinouani et al., 2024[45]), the features of e-cigarettes (Kinouani et al., 2024[45]) or their design (Kava et al., 2021[41]; Kinouani et al., 2024[45]). Some young adults also cited ease of use (Kechter et al., 2022[42]; Kinouani et al., 2024[45]) and pleasure (Robertson et al., 2022[70]; Kinouani et al., 2024[45]) as reasons for use. Some qualitative studies identified several actors who encouraged continued vaping: family or friends who provided the first e-cigarette (Cheney et al., 2016[17]), peers (Robertson et al., 2022[70]) or celebrities (Kava et al., 2021[41]). One study also identified features of student life that might encourage continued use of e-cigarettes: greater perceived freedom, more social events, especially when alcohol use is encouraged (Kava et al., 2021[41]).

As shown in Supplementary information (Table S4), the most common extrinsic factors reported in the articles for continued e-cigarette use were the appeal of e-liquid flavors and the ability to vape where smoking is prohibited. The studies also highlighted the practicality/ease of use, possibility to customize use and social integration or acceptability as extrinsic factors commonly reported to support continued use. Smoking cessation or reduction, less harm and lower cost compared to smoking and seeking the psychotropic effects of nicotine were the most commonly reported intrinsic factors for continued use.

### Robustness analysis

In the second analysis, 16 articles of low global quality assessment (Lotrean, 2015[52]; Dunlop et al., 2016[23]; Martinasek et al., 2018[53]; Hong et al., 2019[35]; Jongenelis et al., 2019[40]; Case et al., 2020[16]; Ickes et al., 2020[39]; Tamulevicius et al., 2020[78]; Newcombe et al., 2021[56]; Chok et al., 2023[19]; Freeman et al., 2023[25]; Obisesan et al., 2023[58]; Stone et al., 2023[76]; Roh et al., 2024[71]; Thoonen and Jongenelis, 2024[79]) or with a conflict of interest statement (Kava et al., 2021[41]) were removed from the systematic review. As shown in Supplementary information (Tables S5 and S6), results were similar to the main analyses regarding the most commonly reported intrinsic, sociocultural or structural reasons for e-cigarette use, whether for its initiation or for its continuation.

## Discussion

In this review, we found that young adults combined intrinsic and extrinsic reasons for using e-cigarettes. A category of extrinsic reasons related to vaping products and their features emerged during our data synthesis. The appeal of e-liquid flavors was one of the reasons from this same category that was very frequently cited as encouraging both experimentation and continued use of e-cigarettes. Quitting smoking or perceiving vaping as less harmful than smoking were the most reported intrinsic reasons for initiating e-cigarette use. Young adults also reported doing so out of curiosity (regardless of their smoking status) or as an opportunity in the presence of other vapers. For continued e-cigarette use, the most reported intrinsic reasons in studies were quitting or reducing smoking, perceived benefits of vaping over smoking (lower cost, less perceived harm), and seeking the psychotropic effects of nicotine. The ability to vape where smoking is prohibited, convenience/ ease of use, ability to customize use, social acceptability, and social facilitation were the most reported as extrinsic reasons for continued use.

We found it was necessary to individualize the extrinsic reasons associated with vaping product features during data synthesis. Other studies have also found that the characteristics of e-liquids and electronic devices promote e-cigarette use (Harvanko et al., 2018[33]; Alqahtani et al., 2022[1]), particularly flavor variety (Huang et al., 2017[38]; Kowitt et al., 2017[47]; Gendall and Hoek 2021[29]). Flavor seems to be very important to young adults (Bonhomme et al., 2016[11]; Cooper et al., 2016[20]; Patel et al., 2016[63]; Buckell and Sindelar 2019[13]; Baker et al., 2021[9]; Whaley et al., 2024[88]), who are more likely than older adults to cite it as a reason for using e-cigarettes (Bonhomme et al., 2016[11]; Cooper et al., 2016[20]; Patel et al., 2016[63]). The role of e-liquid flavors in vaping initiation is even more important to monitor because there is insufficient evidence that they also promote smoking cessation among tobacco smokers (Huang et al., 2017[38]; Zare et al., 2018[91]). Although some studies suggest that non-tobacco flavored e-liquids may promote smoking cessation more than tobacco flavored e-liquids (Brandon et al., 2019[12]; Friedman and Xu, 2020[27]; Gravely et al., 2020[30]; Harlow et al., 2022[32]), the risk-benefit balance remains to be determined. It needs to be confirmed by further studies.

Sociocultural extrinsic factors have previously been described as promoting the use of e-cigarettes among adults (Soule et al., 2016[73]; Wadsworth et al., 2016[87]; Nicksic et al., 2019[57]; Yong et al., 2019[90]; Amin et al., 2020[2]). Our findings suggest that social acceptability is a well-identified factor for maintaining e-cigarette use among young adults, while having e-cigarette users in one's social circle promotes both initiation and continuation of vaping. Conversely, few of the included studies described structural extrinsic reasons as determinants of e-cigarette use, as if young adults did not spontaneously identify them as such. The two main structural reasons described by young adults were the ability to vape where smoking is prohibited and exposure to advertising for vaping products. This is consistent with findings from some studies not limited to young adults (Wadsworth et al., 2016[87]; Kim et al., 2017[44]; Lee et al., 2018[50]; Cheng et al., 2019[18]; Amin et al., 2020[2]). Other studies have shown that e-cigarette regulatory policies subsequently affect e-cigarette or tobacco use among young adults. A longitudinal study of more than 17,000 Americans aged 18 to 24 conducted between 2014 and 2019 found that current e-cigarette use increased overall during this period, but the increase was less rapid in American states with vaping product excise tax policies (Han et al., 2023[31]). Another study showed that increasing taxes on e-cigarettes decreased e-cigarette use among Americans aged 18 to 25, but increased their use of tobacco cigarettes (Friedman and Pesko, 2022[26]). A study was conducted in 2021 among 18-34-year-old Americans who vaped flavored e-liquids. The study explored their attitudes toward a restriction on the sale of flavors (whether this restriction was in effect in their state or hypothetical). In the context of an actual or hypothetical ban on flavors in e-liquids, nearly 80 % said they would continue to vape; 12.5 % of those who exclusively vaped prior to an effective flavor restriction had switched to tobacco products (Tam et al., 2024[77]). Finally, a study conducted simultaneously in Canada, Australia and the UK showed a differential impact of regulating nicotine levels in vaping products on tobacco use among young adults. In most Canadian provinces where higher nicotine levels were allowed in vaping products, the introduction of vaping led to a reduction in smoking prevalence. In the UK where the maximum nicotine level allowed in vaping products was 20 mg/ml, the introduction of vaping slowed the declining trend in cigarette prevalence among men aged 16-34. In Australia, where nicotine was not permitted in e-cigarettes, the introduction of e-cigarettes slowed the declining trend in smoking prevalence among men aged 18-24 years (Wu et al., 2022[89]). These different studies show that policies regulating vaping products influence young adults' use of e-cigarettes, even if they do not realize it. However, it seems relevant to combine several policies regulating vaping products to act on different levers, and also to assess their impact taking into account the evolution of tobacco use.

There is no universal definition of who is a young adult. For psychologists such as Arnett, the transition to adulthood is no longer defined by social events such as marriage or the onset of parenthood since the mid-20th century. This transition is now more dependent on individual psychological development, which begins during adolescence but peaks during a distinct stage of life that Arnett has termed "emerging adulthood" (Arnett, 2000[3], 2024[4]). He initially placed this phase between the ages of 18 and 25 (Arnett, 2000[3]), before later extending it to the period between 18 and 29 (Arnett, 2000[3], 2007[5], 2024[4]; Arnett et al., 2014[7]). Sociologists have made a nearly analogous observation regarding the desynchronization of social markers of transition in the young adult population of industrialized countries. What distinguishes them from psychologists such as Arnett is the emphasis that they place on extrinsic factors in the development of personal trajectories (Van de Velde, 2008[83]; Galland, 2022[28]). Describing the trajectory of e-cigarette use among 18-30-year-olds allows psychologists and sociologists to partially converge. As we hypothesized, both experimentation and continued use of e-cigarettes among young adults seemed to be justified by a combination of extrinsic and intrinsic reasons. Their decision to vape is certainly a personal choice but one that is made possible by the national framework that regulates the sale and use of vaping products, particularly the price and the availability of nicotine or flavors in e-liquids.

This review, likely to be one of the most up-to-date explorations of the reasons for e-cigarette use among young adults, may not be exhaustive. Efforts were made to include a wide range of studies by diversifying database searches, incorporating gray literature, and considering all study designs. Nonetheless, several limitations should be acknowledged. It included many cross-sectional studies conducted on convenience and non-representative samples. Therefore, the performed data synthesis is mainly a qualitative description of the reasons for using e-cigarettes. The generalizability of the findings in low- and middle-income countries is also limited by the preponderance of American studies. Definitions of e-cigarette use varied across the included studies, especially for continuous use. If information was missing during the full reading of the articles, we tried to find this information by reading the protocols or going to the websites of the studies. If the information remained missing, the study was excluded, but we could have contacted the authors of the articles before deciding on their exclusion. The quality of reporting of the articles was assessed by a single reviewer. Finally, the distinction between intrinsic, sociocultural, structural and vaping product feature reasons is fictitious. For example, seeing celebrities vaping on television or in reviews is both a matter of social acceptability and tolerance on the part of policymakers to encourage use through less restrictive regulation.

## Conclusion

Despite its limitations, this research represents the first comprehensive overview of the diverse reasons for e-cigarette use among 18-30-year-olds. It underscores the importance of distinguishing between reasons for initiation and continued use, highlighting that these motivations are varied and often interconnected. Additionally, it demonstrates that young adults do not solely perceive e-cigarettes as a smoking cessation tool. 

To better address this behavior, health professionals should explore the reasons for e-cigarette use through open-ended questions, allowing for a more nuanced understanding of the diverse motivational factors at play. This approach could provide valuable insights into the complex trajectories of e-cigarette use in this population.

While intrinsic reasons were frequently cited in the studies, young adults rarely acknowledged the structural context as a decisive factor. However, this context likely plays a significant role in shaping the motivations for initiating or continuing e-cigarette use, as it is influenced by each country's regulatory policies governing the sale, use, and promotion of tobacco and vaping products. Future research, particularly qualitative studies, should focus on exploring young adults' awareness of, adherence to, or resistance against these regulatory frameworks. This could provide valuable insights into how structural factors influence vaping behavior and how such behavior evolves in response to policy changes.

## Declaration

### Acknowledgments

The authors would like to thank Ms. Hélène Plouseau-Guédé for her assistance in selecting databases. They also thank Ms. Jacqueline Pedley for English language copyediting and Mr. Paul Vanderkam for his advice on the protocol. During the preparation of this work, the authors used DeepL Write® (software with artificial intelligence) to copyedit the manuscript. After using this tool, the authors revised the content as needed and they take full responsibility for the content of the publication.

### Conflict of interest

The authors declare that they have no conflict of interest.

### Authors' contribution

SK, MA, and CT conceptualized the study and drafted the initial manuscript. SK, FR, BQ performed data screening, extraction and analyses. They were also responsible for quality assessment. SK performed data synthesis. All authors approved the final version of the manuscript.

## Supplementary Material

Supplementary information

## Figures and Tables

**Table 1:**
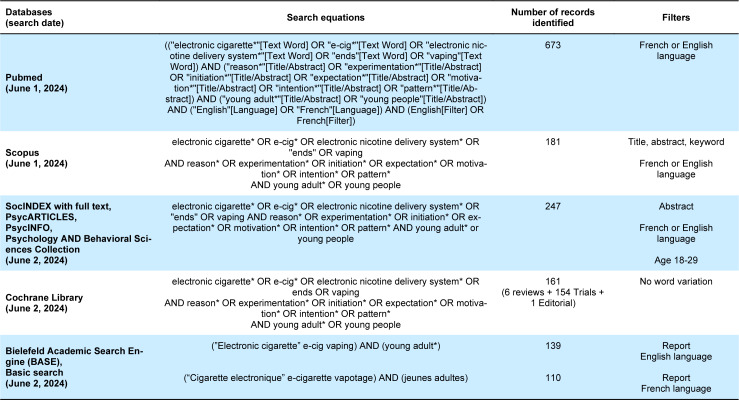
Search strategy

**Table 2:**
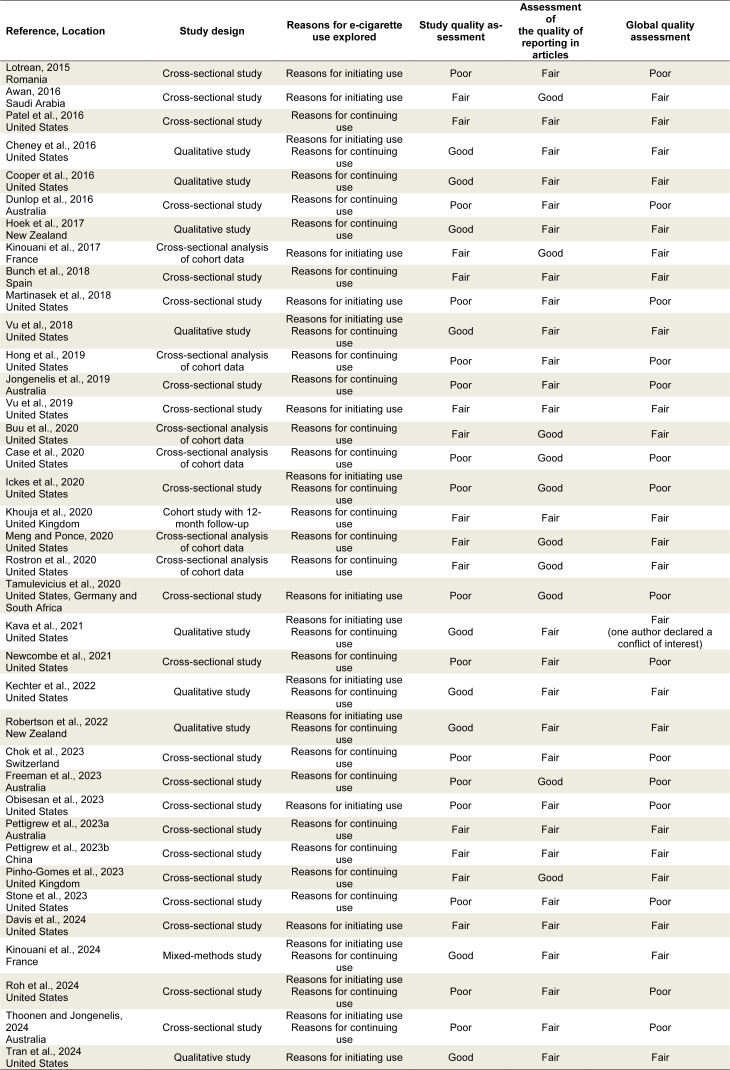
Characteristics of the included studies

**Table 3:**
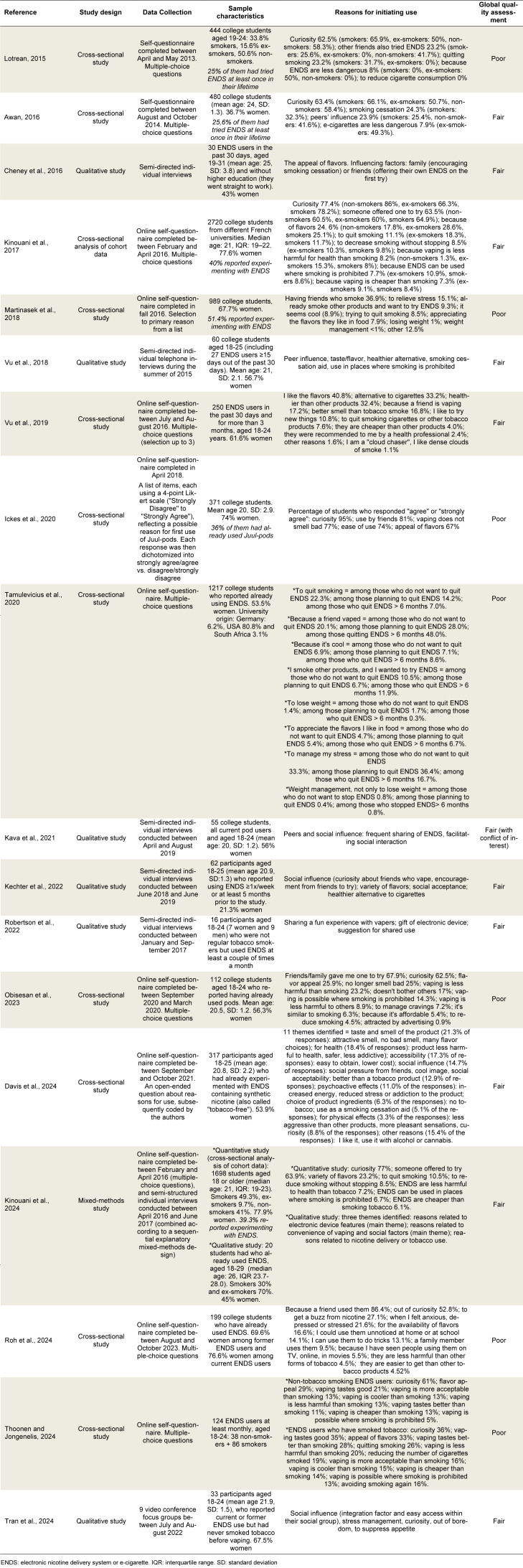
Description of studies examining reasons for initiation of e-cigarette use among 18-30-year-olds

**Table 4:**
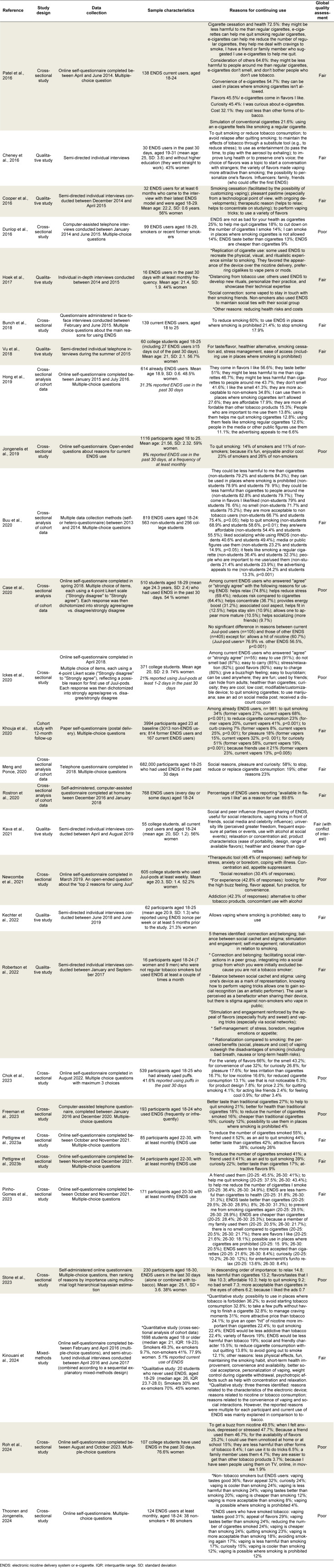
Description of studies examining reasons for continuing use of e-cigarettes among 18-30-year-olds

## References

[1] Alqahtani MM, Massey ZB, Fairman RT, Churchill V, Ashley DL, Popova L (2022). General and device-specific reasons for ENDS use: a qualitative study with adult ENDS users. Int J Environ Res Public Health.

[2] Amin S, Dunn AG, Laranjo L (2020). Social influence in the uptake and use of electronic cigarettes: a systematic review. Am J Prev Med.

[3] Arnett JJ (2000). Emerging adulthood. A theory of development from the late teens through the twenties. Am Psychol.

[4] Arnett JJ (2024). Emerging adulthood: the winding road from the late teens through the twenties.

[5] Arnett JJ (2007). Emerging adulthood: what is it, and what is it good for?. Child Dev Perspect.

[6] Arnett JJ (2005). The developmental context of substance use in emerging adulthood. J Drug Issues.

[7] Arnett JJ, Žukauskienė R, Sugimura K (2014). The new life stage of emerging adulthood at ages 18–29 years: implications for mental health. Lancet Psychiatry.

[8] Awan KH (2016). Experimentation and correlates of electronic nicotine delivery system (electronic cigarettes) among university students – A cross sectional study. Saudi Dent J.

[9] Baker AN, Wilson SJ, Hayes JE (2021). Flavor and product messaging are the two most important drivers of electronic cigarette selection in a choice-based task. Sci Rep.

[10] Becker HS (1963). In:Outsiders; studies in the sociology of deviance.

[11] Bonhomme MG, Holder-Hayes E, Ambrose BK, Tworek C, Feirman SP, King BA (2016). Flavoured non-cigarette tobacco product use among US adults: 2013-2014. Tob Control.

[12] Brandon KO, Simmons VN, Meltzer LR, Drobes DJ, Martínez Ú, Sutton SK (2019). Vaping characteristics and expectancies are associated with smoking cessation propensity among dual users of combustible and electronic cigarettes. Addiction.

[13] Buckell J, Sindelar JL (2019). The impact of flavors, health risks, secondhand smoke and prices on young adults’ cigarette and e-cigarette choices: a discrete choice experiment. Addiction.

[14] Bunch K, Fu M, Ballbè M, Matilla-Santader N, Lidón-Moyano C, Martin-Sanchez JC (2018). Motivation and main flavour of use, use with nicotine and dual use of electronic cigarettes in Barcelona, Spain: a cross-sectional study. BMJ Open.

[15] Buu A, Hu Y-H, Wong S-W, Lin H-C (2020). Comparing American college and noncollege young adults on e-cigarette use patterns including polysubstance use and reasons for using e-cigarettes. J Am Coll Health.

[16] Case KR, Hinds JT, Creamer MR, Loukas A, Perry CL (2020). Who is JUULing and why? An Examination of young adult electronic nicotine delivery systems users. J Adolesc Health.

[17] Cheney MK, Gowin M, Wann TF (2016). Electronic cigarette use in straight-to-work young adults. Am J Health Behav.

[18] Cheng K, Chaloupka FJ, Shang C, Ngo A, Fong GT, Borland R (2019). Prices, use restrictions and electronic cigarette use—evidence from wave 1 (2016) US data of the ITC Four Country Smoking and Vaping Survey. Addiction.

[19] Chok L, Cros J, Lebon L, Zürcher K, Dubuis A, Berthouzoz C (2023). Enquête sur l’usage et les représentations des cigarettes électroniques jetables (puffs) parmi les jeunes romand·es.

[20] Cooper M, Harrell MB, Perry CL (2016). Comparing young adults to older adults in e-cigarette perceptions and motivations for use: implications for health communication. Health Educ Res.

[21] Cornelius ME, Loretan CG, Jamal A, Davis Lynn BC, Mayer M, Alcantara IC (2023). Tobacco product use among adults - United States, 2021. MMWR Morb Mortal Wkly Rep.

[22] Davis DR, Rajesh Kumar L, Morean ME, Kong G, Bold KW, Krishnan-Sarin S (2024). Why young adults use tobacco-free nicotine E-cigarettes: An analysis of qualitative data. Addict Behav.

[23] Dunlop S, Lyons C, Dessaix A, Currow D (2016). How are tobacco smokers using e-cigarettes? Patterns of use, reasons for use and places of purchase in New South Wales. Med J Aust.

[24] Foxon F, Selya A, Gitchell J, Shiffman S (2024). Increased e-cigarette use prevalence is associated with decreased smoking prevalence among US adults. Harm Reduct J.

[25] Freeman B, Owen K, Rickards S, Brooks A, Clare PJ, Dessaix A (2023). E-cigarette use by people who smoke or have recently quit, New South Wales, 2016–2020. Med J Aust.

[26] Friedman AS, Pesko MF (2022). Young adult responses to taxes on cigarettes and electronic nicotine delivery systems. Addiction.

[27] Friedman AS, Xu S (2020). Associations of flavored e-cigarette uptake with subsequent smoking initiation and cessation. JAMA Netw Open.

[28] Galland O (2022). Sociologie de la jeunesse.

[29] Gendall P, Hoek J (2021). Role of flavours in vaping uptake and cessation among New Zealand smokers and non-smokers: a cross-sectional study. Tob Control.

[30] Gravely S, Cummings KM, Hammond D, Lindblom E, Smith DM, Martin N (2020). The association of E-cigarette flavors with satisfaction, enjoyment, and trying to quit or stay abstinent from smoking among regular adult vapers from canada and the United States: Findings from the 2018 ITC Four Country Smoking and Vaping Survey. Nicotine Tob Res.

[31] Han D-H, Seo D-C, Lin H-C (2023). Statewide vaping product excise tax policy and use of electronic nicotine delivery systems among US young adults, 2014–2019. Tob Control.

[32] Harlow AF, Fetterman JL, Ross CS, Robertson RM, Bhatnagar A, Benjamin EJ (2022). Association of device type, flavours and vaping behaviour with tobacco product transitions among adult electronic cigarette users in the USA. Tob Control.

[33] Harvanko AM, McCubbin AK, Ashford KB, Kelly TH (2018). Electronic cigarette liquid and device parameters and aerosol characteristics: A survey of regular users. Addict Behav.

[34] Hoek J, Thrul J, Ling P (2017). Qualitative analysis of young adult ENDS users’ expectations and experiences. BMJ Open.

[35] Hong H, McConnell R, Liu F, Urman R, Barrington-Trimis JL (2019). The impact of local regulation on reasons for electronic cigarette use among Southern California young adults. Addict Behav.

[36] Hong QN, Gonzalez-Reyes A, Pluye P (2018). Improving the usefulness of a tool for appraising the quality of qualitative, quantitative and mixed methods studies, the Mixed Methods Appraisal Tool (MMAT). J Eval Clin Pract.

[37] Hong QN, Pluye P, Fàbregues S, Bartlett G, Boardman F, Cargo M (2019). Improving the content validity of the mixed methods appraisal tool: a modified e-Delphi study. J Clin Epidemiol.

[38] Huang L-L, Baker HM, Meernik C, Ranney LM, Richardson A, Goldstein AO (2017). Impact of non-menthol flavours in tobacco products on perceptions and use among youth, young adults and adults: a systematic review. Tob Control.

[39] Ickes M, Hester JW, Wiggins AT, Rayens MK, Hahn EJ, Kavuluru R (2020). Prevalence and reasons for Juul use among college students. J Am Coll Health.

[40] Jongenelis MI, Brennan E, Slevin T, Kameron C, Rudaizky D, Pettigrew S (2019). Differences in use of electronic nicotine delivery systems by smoking status and demographic characteristics among Australian young adults. Health Promot J Austr.

[41] Kava CM, Soule EK, Seegmiller L, Gold E, Snipes W, Westfield T (2021). “Taking up a new problem” - Context and determinants of pod-mod e-cigarette use among college students. Qual Health Res.

[42] Kechter A, Simpson KA, Ceasar RC, Schiff SJ, Yamaguchi N, Bluthenthal RN (2022). Trajectories of nicotine use leading to dual and cyclical tobacco product use in young adults. Nicotine Tob Res.

[43] Khouja JN, Taylor AE, Munafò MR (2020). Associations between reasons for vaping and current vaping and smoking status: Evidence from a UK based cohort. Drug Alcohol Depend.

[44] Kim H, Davis AH, Dohack JL, Clark PI (2017). E-Cigarettes use behavior and experience of adults: qualitative research findings to inform e-cigarette use measure development. Nicotine Tob Res.

[45] Kinouani S, Da Cruz H, Langlois E, Tzourio C (2024). Prevalence, lived experiences and user profiles in e-cigarette use: A mixed methods study among French college students. PLoS ONE.

[46] Kinouani S, Pereira E, Tzourio C (2017). Electronic cigarette use in students and its relation with tobacco-smoking: a cross-sectional analysis of the i-share study. Int J Environ Res Public Health.

[47] Kowitt SD, Meernik C, Baker HM, Osman A, Huang L-L, Goldstein AO (2017). Perceptions and experiences with flavored non-menthol tobacco products: a systematic review of qualitative studies. Int J Environ Res Public Health.

[48] Kramarow EA, Elgaddal N (2023). Current electronic cigarette use among adults aged 18 and over: United States, 2021. NCHS Data Brief.

[49] Laverty AA, Filippidis FT, Vardavas CI (2018). Patterns, trends and determinants of e-cigarette use in 28 European Union Member States 2014–2017. Prev Med.

[50] Lee C, Yong H-H, Borland R, McNeill A, Hitchman SC (2018). Acceptance and patterns of personal vaporizer use in Australia and the United Kingdom: Results from the International Tobacco Control survey. Drug Alcohol Depend.

[51] Levitt HM, Bamberg M, Creswell JW, Frost DM, Josselson R, Suárez-Orozco C (2018). Journal article reporting standards for qualitative primary, qualitative meta-analytic, and mixed methods research in psychology: The APA Publications and Communications Board task force report. Am Psychol.

[52] Lotrean LM (2015). Use of electronic cigarettes among Romanian university students: a cross-sectional study. BMC Public Health.

[53] Martinasek MP, Bowersock A, Wheldon CW (2018). Patterns, Perception and behavior of electronic nicotine delivery systems use and multiple product use among young adults. Respir Care.

[54] McKeganey N, Barnard M, Russell C (2018). Vapers and vaping: E-cigarettes users views of vaping and smoking. Drugs Educ Prev Policy.

[55] Meng Y-Y, Ponce NA (2020). The changing landscape: tobacco and marijuana use among young adults in California.

[56] Newcombe KV, Dobbs PD, Oehlers JS, Dunlap CM, Cheney MK (2021). College students’ reasons for using JUULs. Am J Health Promot.

[57] Nicksic NE, Snell LM, Barnes AJ (2019). Reasons to use e-cigarettes among adults and youth in the Population Assessment of Tobacco and Health (PATH) study. Addict Behav.

[58] Obisesan OH, Uddin SMI, Boakye E, Osei AD, Mirbolouk M, Orimoloye OA (2023). Pod-based e-cigarette use among US college-aged adults: A survey on the perception of health effects, sociodemographic correlates, and interplay with other tobacco products. Tob Induc Dis.

[59] OECD/European Union (2022). Health at a glance: Europe 2022. State of health in the EU cycle. https://www.oecd-ilibrary.org/docserver/507433b0-en.pdf?expires=1725395594&id=id&accname=guest&checksum=CBE32A7E14AE43A4A5FBEEC186AEF294.

[60] Ouzzani M, Hammady H, Fedorowicz Z, Elmagarmid A (2016). Rayyan—a web and mobile app for systematic reviews. Syst Rev.

[61] Pace R, Pluye P, Bartlett G, Macaulay AC, Salsberg J, Jagosh J (2012). Testing the reliability and efficiency of the pilot Mixed Methods Appraisal Tool (MMAT) for systematic mixed studies review. Int J Nurs Stud.

[62] Page MJ, McKenzie JE, Bossuyt PM, Boutron I, Hoffmann TC, Mulrow CD (2021). The PRISMA 2020 statement: an updated guideline for reporting systematic reviews. BMJ.

[63] Patel D, Davis KC, Cox S, Bradfield B, King BA, Shafer P (2016). Reasons for current E-cigarette use among U.S. adults. Prev Med.

[64] Peretti-Watel P, Beck F, Legleye S, Moatti J-P (2007). Becoming a smoker: Adapting Becker’s model of deviance for adolescent smoking. Health Sociol Rev.

[65] Peretti-Watel P, Beck, F, Legleye S (2007). Les usages sociaux des drogues.

[66] Peretti-Watel P, Halfen S, Grémy I (2007). The ‘moral career’ of cigarette smokers: A French survey. Health Risk Soc.

[67] Pettigrew S, Miller M, Alvin Santos J, Raj TS, Brown K, Jones A (2023). E-cigarette attitudes and use in a sample of Australians aged 15–30 years. Aust N Z J Public Health.

[68] Pettigrew S, Santos JA, Li Y, Miller M, Anderson C, Raj TS (2023). E-cigarette-related beliefs, behaviors, and policy support among young people in China. Tob Induc Dis.

[69] Pinho-Gomes A-C, Santos JA, Jones A, Thout SR, Pettigrew S (2023). E-cigarette attitudes and behaviours amongst 15-30-year-olds in the UK. J Public Health.

[70] Robertson L, Hoek J, Blank M-L (2022). A qualitative analysis of electronic nicotine delivery systems (ENDS) uptake and use among young adult never-smokers in New Zealand. PLoS ONE.

[71] Roh T, Fields S, Sahu R, Trisha NF, Carrillo G (2024). Vaping behavior and intention to quit among undergraduate students in a hispanic-serving university. J Community Health.

[72] Rostron BL, Yu-Ching Cheng, Gardner LD, Ambrose BK (2020). Prevalence and reasons for use of flavored cigars and ENDS among US youth and adults: estimates from wave 4 of the PATH Study, 2016-2017. Am J Health Behav.

[73] Soule EK, Rosas SR, Nasim A (2016). Reasons for electronic cigarette use beyond cigarette smoking cessation: A concept mapping approach. Addict Behav.

[74] Souto RQ, Khanassov V, Hong QN, Bush PL, Vedel I, Pluye P (2015). Systematic mixed studies reviews: Updating results on the reliability and efficiency of the mixed methods appraisal tool. Int J Nurs Stud.

[75] Steinmetz-Wood M, Gagné T, Sylvestre M-P, Frohlich K (2018). Do social characteristics influence smoking uptake and cessation during young adulthood?. Int J Public Health.

[76] Stone MD, Braymiller JL, Strong DR, Cwalina SN, Dimofte CV, Barrington-Trimis JL (2023). Differentiating reasons for young adult e-cigarette use using maximum difference choice models. Nicotine Tob Res.

[77] Tam J, Jimenez-Mendoza E, Buckell J, Sindelar J, Meza R (2024). Responses to real-world and hypothetical e-cigarette flavor bans among US Young adults who use flavored E-cigarettes. Nicotine Tob Res.

[78] Tamulevicius N, Martinasek MP, Moss SJ, Pfeffer I, Gibson-Young LM, Yahaya M (2020). An analysis of associations between electronic nicotine delivery system users. Respir Care.

[79] Thoonen KAHJ, Jongenelis MI (2024). Motivators of e-cigarette use among Australian adolescents, young adults, and adults. Soc Sci Med.

[80] Tong A, Sainsbury P, Craig J (2007). Consolidated criteria for reporting qualitative research (COREQ): a 32-item checklist for interviews and focus groups. Int J Qual Health Care.

[81] Tran DD, Davis JP, Ring C, Buch K, Fitzke RE, Pedersen ER (2024). A deeper dive into young adults’ experiences with E-cigarettes, E-cigarette cessation, and transitioning to cigarette smoking. Subst Use Misuse.

[82] Urman R, McConnell R, Unger JB, Cruz TB, Samet JM, Berhane K (2019). Electronic cigarette and cigarette social environments and ever use of each product: a prospective study of young adults in southern California. Nicotine Tob Res.

[83] Van de Velde C (2008). Devenir adulte: sociologie comparée de la jeunesse en Europe.

[84] von Elm E, Altman DG, Egger M, Pocock SJ, Gøtzsche PC, Vandenbroucke JP (2007). The Strengthening the Reporting of Observational Studies in Epidemiology (STROBE) statement: guidelines for reporting observational studies. PLoS Med.

[85] Vu M, Getachew B, Payne JB, Kirchner TR, Berg CJ (2018). Initiation, continuation of use and cessation of alternative tobacco products among young adults: A qualitative study. Tob Prev Cessat.

[86] Vu T-HT, Hart JL, Groom A, Landry RL, Walker KL, Giachello AL (2019). Age differences in electronic nicotine delivery systems (ENDS) usage motivations and behaviors, perceived health benefit, and intention to quit. Addict Behav.

[87] Wadsworth E, Neale J, McNeill A, Hitchman SC (2016). How and why do smokers start using E-cigarettes? qualitative study of vapers in London, UK. Int J Environ Res Public Health.

[88] Whaley RC, Harlow AF, Krueger EA, Stone MD, Dimofte CV, Strong DR (2024). Importance of various E-cigarette device and E-liquid characteristics by smoking status among young adults who vape. Subst Use Misuse.

[89] Wu DC, Essue BM, Jha P (2022). Impact of vaping introduction on cigarette smoking in six jurisdictions with varied regulatory approaches to vaping: an interrupted time series analysis. BMJ Open.

[90] Yong H-H, Borland R, Cummings KM, Gravely S, Thrasher JF, McNeill A (2019). Reasons for regular vaping and for its discontinuation among smokers and recent ex-smokers: findings from the 2016 ITC Four Country Smoking and Vaping Survey. Addiction.

[91] Zare S, Nemati M, Zheng Y (2018). A systematic review of consumer preference for e-cigarette attributes: Flavor, nicotine strength, and type. PLoS ONE.

