# Supplementary material for: Reasons for using electronic cigarettes among young adults aged 18 – 30: a systematic review
Source: EXCLI J. 2025 Jan 31;24:204–37. doi: 10.17179/excli2024-8085 (PMC11895060; doi:10.17179/excli2024-8085)
Supplement: Supplementary information [file EXCLI-24-204-s-001.pdf]

EXCLI Journal 2025;24:204-237 – ISSN 1611-2156
Received: December 07, 2024, accepted: January 27, 2025, published: January 31, 2025

Supplementary information to:

Review article:

REASONS FOR USING ELECTRONIC CIGARETTES AMONG YOUNG ADULTS AGED 18 – 30: A SYSTEMATIC REVIEW

Shérazade Kinouani[1,2*], Faustine Roux[2], Bastien Questel[2], Maëlys Abraham[3], Christophe Tzourio[1]

[1] University of Bordeaux, Inserm, Bordeaux Population Health Research Center, team HEALTHY, UMR 1219, Bordeaux, France
[2] Department of General Practice, University of Bordeaux, Bordeaux, France
[3] 48 avenue de Labarde, Bordeaux, France

[*] **Corresponding author:** Shérazade Kinouani, Centre INSERM U1219 BPH, Equipe HEALTHY, Université de Bordeaux, 146 rue Léo Saignat, CS61292, 33076 Bordeaux Cedex France. Tel: + 33 (0)6 10 29 68 47. E-mail: sherazade.kinouani@u-bordeaux.fr

https://dx.doi.org/10.17179/excli2024-8085

This is an Open Access article distributed under the terms of the Creative Commons Attribution License (http://creativecommons.org/licenses/by/4.0/).

EXCLI Journal 2025;24:204-237 – ISSN 1611-2156
Received: December 07, 2024, accepted: January 27, 2025, published: January 31, 2025

Table S1: Global quality assessment

		Quality assessment of reporting in the article (All articles assessed by SK)		
		**Poor**	**Fair**	**Good**
**Study quality assessment**<br>• 18 articles by FR and SK<br>• 19 articles by BQ and SK	**Poor**	Poor	Poor	Poor
	**Fair**	Poor	Fair	Fair
	**Good**	Fair	Fair	Good

BQ: Bastien Questel; FR: Faustine Roux; SK: Shérazade Kinouani

EXCLI Journal 2025;24:204-237 – ISSN 1611-2156
Received: December 07, 2024, accepted: January 27, 2025, published: January 31, 2025

Table S2: Definitions of reasons for using e-cigarettes

Reasons for using e-cigarettes	Different definitions used	References
Reasons for initiating use	Have tried an e-cigarette at least once	(1–4)
	Have already used an e-cigarette, even for just 1 (or 2) puffs	(5–8)
	Starting to use an e-cigarette	(9)
	First use of e-cigarette	(10–14)
	First time (or first experience) vaping	(15,16)
	Initiation of e-cigarette use	(17,18)
Reasons for continuing use	Use in last month	(8,10,19)
	Use e-cigarettes at least monthly	(20)
	Use of e-cigarettes (at least once) in the past 30 days	(14,21–25)
	Use of an e-cigarette at least once a month, with at least one used in the past 30 days	(26–28)
	Use of e-cigarettes at least 1-2 days in the past 30 days	(5)
	Use of e-cigarettes at least twice a month	(13)
	Use of e-cigarettes at least twice a week on average in the past month	(15)
	Use of e-cigarettes over at least 15 days in the past 30 days	(17)

	Having your own e-cigarette and using it at least weekly	(29)
	Frequent (daily or weekly) use of e-cigarettes	(30)
	Weekly use of e-cigarettes for at least the past 5 months	(16)
	Have used e-cigarettes for at least 6 months and come to the interview with your latest e-cigarette model	(31)
	Current e-cigarette use	(32–34)
	Current use of e-cigarettes, whether occasional or daily	(3,35–37)

EXCLI Journal 2025;24:204-237 – ISSN 1611-2156
Received: December 07, 2024, accepted: January 27, 2025, published: January 31, 2025

Table S3: Reasons for initiating e-cigarette use among adults aged 18-30 categorized into intrinsic, sociocultural and structural factors

**INTRINSIC FACTORS**	**EXTRINSIC FACTORS**		
	**SOCIOCULTURAL FACTORS**	**STRUCTURAL FACTORS**	**FACTORS RELATED TO ELECTRONIC DEVICES AND E-LIQUIDS**
	Influential actors: family, friends, peers, celebrities, healthcare professionals		
**Curiosity**	**Factors related to the human environment**	**Healthcare stakeholder positioning**	• *Flavor appeal* (2,3,5–12,16,17)
• *Curiosity* (1–5,7–9,14,16,18)	• *Close circle who have already used ENDS* (1,3–6,10,12,14,16,17)	• *Recommended by a healthcare profes-sional* (12)	• *Ease of use* (3,5)
**Pleasant practice**	• *Family strongly encourages smoking cessation* (10)	**Regulation of use**	• *Discretion of use* (3,14)
• *Vaping tricks* (3,12,14)	• *Someone offered to try* (2,3,9,10,13,15)	• *Vaping in places where smoking is banned* (2,3,8,9,17)	
• *Practice found enjoya-ble* (3,7,13)	• *Social integration, facilitation of social interactions among peers* (3,15,18)	**Sales regulations**	
**Self-image**	**Social acceptability**	• *ENDS and e-liquids availability* (7,14)	
Cool practice (6–8,11)	• *Social acceptability* (7,8,16)	**E-cigarette visibility/Adver-tising regulations**	

**Vaping compared to smoking**		• *Attractiveness of ads* (9)	
• *Lower cost* (2,3,7,8,12)		• *Celebrities (on TV or online) who use ENDS* (14)	
• *Less harmfulness* (1–4,7–9,12,14,16,17)			
• *Less (bad) smell* (5,7–9,12)			
• *Similarity to smoking* (3,9)			
**Managing tobacco use**			
• *Smoking cessation* (1–4,6–8,11,12,17)			
• *Reducing tobacco use* (2,3,8,9)			
• *Reducing exposure to second-hand smoke* (3,9)			
**Managing tobacco use disorder**			
• *Managing cravings* (7,9)			

• *To avoid relapsing to smoking* (8)			
Nicotine effect expectations			
• *Dealing with stress or anxiety* (7,11,14,18)			
• *Appetite and weight control* (6,11,18)			
• *To find energy gain* (7)			
**Others**			
• *Managing boredom* (18)			
• *Concomitant use with alcohol or cannabis* (7)			

ENDS: electronic nicotine delivery system or e-cigarette

EXCLI Journal 2025;24:204-237 – ISSN 1611-2156
Received: December 07, 2024, accepted: January 27, 2025, published: January 31, 2025

Table S4: Reasons for continuing e-cigarette use among adults aged 18-30 categorized into intrinsic, sociocultural and structural factors

INTRINSIC FACTORS	EXTRINSIC FACTORS		
	SOCIOCULTURAL FACTORS	STRUCTURAL FACTORS	FACTORS RELATED TO ELECTRONIC DEVICES AND E-LIQUIDS
	Influential actors: family, friends, peers, celebrities		
**Curiosity**	**Factors related to the human environment**	**Regulation of use**	• *Flavor appeal* (3,5,8,10,13–15,17,20,21,24,25,27–29,31,35–37)
• *Curiosity* (5,8,20,23,25,27,28,30,34,37)	• *Close circle who have already used ENDS* (5,14,20,21,25,27,28,34,36)	• *Vaping in places where smoking is banned* (3,5,8,16,17,20,21,30,32,33,36–37)	• *Practicality/ease of use* (3,5,16,25,29), *with ease of transport* (5), *or refill* (5)
**Pleasant practice**	• *Social integration, facilitating social interactions among peers* (3,10,13,15,19,22,23,29,36)	• *To avoid going out to smoke* (3)	• *Discretion of use* (3,5,14,25)
• *Vaping tricks* (10,13–15,31)	**Social acceptability**	**Sales regulations**	• *Personalization of use* (3,5,10,15,25,31)
• *Practice found enjoyable and fun* (8,10,20,21,27,28,30,32)	• *Social acceptability* (3,8,20,21,24,36,37)	• *Easy purchase* (5,14)	• *To "Do It Yourself"* (3)

• *Pleasure* (3,13)		• *To receive a discount coupon* (5)	
• *Developing new behavior* (3,19)		**E-cigarette visibility/Advertising regulations**	
• *Developing an expertise* (3,19)		• *Attractiveness of ads* (5,21,24,36)	
**Self-image**		• *Public or media figures who vape* (14,15,21,36)	
• *To appear cool* (3,5,8,22,25,26)			
• *To appear mature* (22)			
• *To use the personal electronic device as a marker of identity* (3,13)			
**Vaping compared to smoking**			
• *Lower cost* (3,5,8,13,19–21,24,25,30,32,36,37)			
• *Better taste* (8,20,21,27,28,30,32)			
• *Less harmfulness* (3,5,8,14,15,17,20,21,24,25,30,32,36–37)			

EXCLI Journal 2025;24:204-237 – ISSN 1611-2156
Received: December 07, 2024, accepted: January 27, 2025, published: January 31, 2025

• *Better smell or no bad smell* (3,5,20,21,24,25,36,37)			
• *Lower perceived addictiveness* (3)			
• *Less disruptive to those around them* (3)			
• *To maintain smoking rituals or rhythms* (3,19)			
• *Similarity to smoking* (3,21,36,37)			
**Managing tobacco use**			
• *Smoking cessation* (3,5,8,10,17,20,21,23–28,30,32–34,36–37)			
• *Reducing tobacco use* (3,8,10,20,23,25,27,28,30,32–34,37)			
• *Health improvement, health risk reduction* (3,10,13,15,19,22)			
• *To take a few puffs without having to finish a cigarette*			

• *Preventing smoking initiation* (3)			
**Managing tobacco use disorder**			
• *Managing cravings* (3,34,37) *or withdrawal symptoms* (3)			
• *To avoid relapsing to smoking* (3,8,10,20)			
**Nicotine effect expectations**			
• *Dealing with stress, anxiety, or depression/ improving concentration* (3,5,10,13–15,17,20,22,24,29,31,37)			
• *Appetite and weight control* (3,13,22,29)			
• *To find energy gain* (5,22,29)			
• *To get a hit of nicotine* (3,14,22)			
• *Managing negative emotions* (13)			

EXCLI Journal 2025;24:204-237 – ISSN 1611-2156
Received: December 07, 2024, accepted: January 27, 2025, published: January 31, 2025

• *Relieving chronic disease symptoms (headaches)* (29)			
**Others**			
• *Managing boredom* (10,13)			
• *Concomitant use with alcohol* (29)			

ENDS: electronic nicotine delivery system or e-cigarette

Table S5: Reasons for initiating e-cigarette use among adults aged 18-30 categorized into intrinsic, sociocultural and structural factors in robustness analysis

INTRINSIC FACTORS	EXTRINSIC FACTORS		
	**SOCIOCULTURAL FACTORS**	**STRUCTURAL FACTORS**	**FACTORS RELATED TO ELECTRONIC DEVICES AND E-LIQUIDS**
	Influential actors: family, friends, peers, healthcare professionals		
**Curiosity**	**Factors related to the human environment**	**Healthcare stakeholder positioning**	• *Flavor appeal* (2,3,7,10–12,16,17)
• *Curiosity* (2–4,7,16,18)	• *Close circle who have already used ENDS* (3,4,10,12,16,17)	• *Recommended by a healthcare professional* (12)	• *Ease of use* (3)
**Pleasant practice**	• *Family strongly encourages smoking cessation* (10)	**Regulation of use**	• *Discretion of use* (3)
• *Vaping tricks* (3,12)	• *Someone offered to try* (2,3,10,13)	• *Vaping in places where smoking is banned* (2,3,17)	
• *Practice found enjoyable* (3,7,13)	*Social integration, facilitating social interactions among peers* (3,15,18)	**Sales regulations**	
**Self-image**	**Social acceptability**	• *ENDS and e-liquids availability* (7)	
• *Cool practice* (7)	• *Social acceptability* (7,16)	**E-cigarette visibility/Advertising regulations**	
**Vaping compared to smoking**		• *Attractiveness of ads*	

• *Lower cost* (2,3,7,12)		• *Celebrities (on TV or online) who use ENDS*	
• *Less harmfulness* (2–4,7,12,16,17)			
• *Less (bad) smell* (7,12)			
• *Similarity to smoking* (3)			
**Managing tobacco use**			
• *Smoking cessation* (2–4,7,12,17)			
• *Reducing tobacco use* (2,3)			
• *Reducing exposure to second-hand smoke* (3)			
**Managing tobacco use disorder**			
• *Managing cravings* (7)			
• *To avoid relapsing to smoking*			
**Nicotine effect expectations**			
• *Dealing with stress or anxiety* (7,18)			

EXCLI Journal 2025;24:204-237 – ISSN 1611-2156
Received: December 07, 2024, accepted: January 27, 2025, published: January 31, 2025

• *Appetite and weight control* (18)			
• *To find energy gain* (7)			
**Others**			
• *Managing boredom* (18)			
• *Concomitant use with alcohol or cannabis* (7)			

ENDS: electronic nicotine delivery system or e-cigarette

EXCLI Journal 2025;24:204-237 – ISSN 1611-2156
Received: December 07, 2024, accepted: January 27, 2025, published: January 31, 2025

Table S6: Reasons for continuing e-cigarette use among adults aged 18-30 categorized into intrinsic, sociocultural and structural factors in robustness analysis

**INTRINSIC FACTORS**	**EXTRINSIC FACTORS**		
	**SOCIOCULTURAL FACTORS**	**STRUCTURAL FACTORS**	**FACTORS RELATED TO ELECTRONIC DEVICES AND E-LIQUIDS**
	Influential actors: family, friends, peers, celebrities		
**Curiosity**	**Factors related to the human environment**	**Regulation of use**	• *Flavor appeal* (3,10,13,17,20,27,28,31,35–37)
• *Curiosity* (20,23,27,28,34,37)	• *Close circle who have already used ENDS* (20,27,28,34,36)	• *Vaping in places where smoking is banned* (3,16,17,20,33,36–37)	• *Practicality/ease of use* (3,16), *with ease of transport or refill*
**Pleasant practice**	• *Social integration, facilitating social interactions among peers* (3,10,13,19,23,36)	• *To avoid going out to smoke* (3)	• *Discretion of use* (3)
• *Vaping tricks* (10,13,31)	**Social acceptability**	**Sales regulations**	• *Personalization of use* (3,10,31)
• *Practice found enjoyable and fun* (10,20,27,28)	• *Social acceptability* (3,20,36,37)	• *For ease of purchase*	• *To “Do It Yourself”* (3)

EXCLI Journal 2025;24:204-237 – ISSN 1611-2156
Received: December 07, 2024, accepted: January 27, 2025, published: January 31, 2025

• *Pleasure* (3,13)		• *To receive a discount coupon*	
• *Developing new behavior* (3,19)		**E-cigarette visibility/Advertising regulations**	
• *Developing an expertise* (3,19)		• *Attractiveness of ads* (36)	
**Self-image**		• *Public or media figures who vape* (36)	
• *To appear cool* (3)			
• *To appear mature*			
• *To use the personal electronic device as a marker of identity* (3,13)			
**Vaping compared to smoking**			
• *Lower cost* (3,13,19,20,36,37)			
• *Better taste* (20,27,28)			
• *Less harmfulness* (3,17,20,36–37)			
• *Better smell or no bad smell* (3,20,36,37)			

• *Lower perceived addictiveness* (3)			
• *Less disruptive to those around them* (3)			
• *To maintain smoking rituals or rhythms* (3,19)			
• *Similarity to smoking* (3,36,37)			
**Managing tobacco use**			
• *Smoking cessation* (3,10,17,20,23,27,28,33,34,36–37)			
• *Reducing tobacco use* (3,10,20,23,27,28,33,34,37)			
• *Health improvement, health risk reduction* (3,10,13,19)			
• *To take a few puffs without having to finish a cigarette* (3)			
• *Preventing smoking initiation* (3)			
Managing tobacco use disorder			

• *Managing cravings* (3,34,37) *or withdrawal symptoms* (3)			
• *To avoid relapsing to smoking* (3,10,20)			
**Nicotine effect expectations**			
Dealing with stress, anxiety, or depression/improving concentration (3,10,13,17,20,31,37)			
Appetite and weight control (3,13)			
To find energy gain			
To get a hit of nicotine (3)			
Managing negative emotions (13)			
Relieving chronic disease symptoms (headaches)			
**Others**			
Managing boredom (10,13)			
Concomitant use with alcohol			

ENDS: electronic nicotine delivery system or e-cigarette

EXCLI Journal 2025;24:204-237 – ISSN 1611-2156
Received: December 07, 2024, accepted: January 27, 2025, published: January 31, 2025

References

1. Lotrean LM. Use of electronic cigarettes among Romanian university students: a cross-sectional study. BMC Public Health. 2015;15(1):358.
2. Kinouani S, Pereira E, Tzourio C. Electronic cigarette use in students and its relation with tobacco-smoking: a cross-sectional analysis of the i-share study. Int J Environ Res Public Health. 2017;14(11):1345.
3. Kinouani S, Da Cruz H, Langlois E, Tzourio C. Prevalence, lived experiences and user profiles in e-cigarette use: A mixed methods study among French college students. PLoS ONE. 2024;19(2):e0297156.
4. Awan KH. Experimentation and correlates of electronic nicotine delivery system (electronic cigarettes) among university students – A cross sectional study. Saudi Dent J. 2016;28(2):91–5.
5. Ickes M, Hester JW, Wiggins AT, Rayens MK, Hahn EJ, Kavuluru R. Prevalence and reasons for Juul use among college students. J Am Coll Health. 2020;68(5):455–9.
6. Tamulevicius N, Martinasek MP, Moss SJ, Pfeffer I, Gibson-Young LM, Yahaya M. An analysis of associations between electronic nicotine delivery system users. Respir Care. 2020;65:355–61.
7. Davis DR, Rajesh Kumar L, Morean ME, Kong G, Bold KW, Krishnan-Sarin S, et al. Why young adults use tobacco-free nicotine E-cigarettes: An analysis of qualitative data. Addict Behav. 2024;150:107925.
8. Thoonen KAHJ, Jongenelis MI. Motivators of e-cigarette use among Australian adolescents, young adults, and adults. Soc Sci Med. 2024;340:116411.
9. Obisesan OH, Uddin SMI, Boakye E, Osei AD, Mirbolouk M, Orimoloye OA, et al. Pod-based e-cigarette use among US college-aged adults: A survey on the perception of health effects, sociodemographic correlates, and interplay with other tobacco products. Tob Induc Dis. 2023;21:34.
10. Cheney MK, Gowin M, Wann TF. Electronic cigarette use in straight-to-work young adults. Am J Health Behav. 2016;40:268–79.
11. Martinasek MP, Bowersock A, Wheldon CW. Patterns, Perception and behavior of electronic nicotine delivery systems use and multiple product use among young adults. Respir Care. 2018;63:913–9.
12. Vu THT, Hart JL, Groom A, Landry RL, Walker KL, Giachello AL, et al. Age differences in electronic nicotine delivery systems (ENDS) usage motivations and behaviors, perceived health benefit, and intention to quit. Addict Behav. 2019;98:106054.
13. Robertson L, Hoek J, Blank ML. A qualitative analysis of electronic nicotine delivery systems (ENDS) uptake and use among young adult never-smokers in New Zealand. PLoS ONE. 2022;17(5):e0268449.
14. Roh T, Fields S, Sahu R, Trisha NF, Carrillo G. Vaping behavior and intention to quit among undergraduate students in a Hispanic-serving university. J Community Health. 2024;49: 820–8.
15. Kava CM, Soule EK, Seegmiller L, Gold E, Snipes W, Westfield T, et al. “Taking up a new problem” - Context and determinants of pod-mod e-cigarette use among college students. Qual Health Res. 2021;31:703–12.
16. Kechter A, Simpson KA, Ceasar RC, Schiff SJ, Yamaguchi N, Bluthenthal RN, et al. Trajectories of nicotine use leading to dual and cyclical tobacco product use in young adults. Nicotine Tob Res. 2022;24:986–93.

EXCLI Journal 2025;24:204-237 – ISSN 1611-2156
Received: December 07, 2024, accepted: January 27, 2025, published: January 31, 2025

17. Vu M, Getachew B, Payne JB, Kirchner TR, Berg CJ. Initiation, continuation of use and cessation of alternative tobacco products among young adults: A qualitative study. Tob Prev Cessat. 2018;4:8.
18. Tran DD, Davis JP, Ring C, Buch K, Fitzke RE, Pedersen ER. A Deeper dive into young adults' experiences with e-cigarettes, e-cigarette cessation, and transitioning to cigarette smoking. Subst Use Misuse. 2024;59:937–46.
19. Hoek J, Thrul J, Ling P. Qualitative analysis of young adult ENDS users' expectations and experiences. BMJ Open. 2017;7(3):e014990.
20. Pinho-Gomes AC, Santos JA, Jones A, Thout SR, Pettigrew S. E-cigarette attitudes and behaviours amongst 15-30-year-olds in the UK. J Public Health. 2023;45:e763–75.
21. Hong H, McConnell R, Liu F, Urman R, Barrington-Trimis JL. The impact of local regulation on reasons for electronic cigarette use among Southern California young adults. Addict Behav. 2019;91:253–8.
22. Case KR, Hinds JT, Creamer MR, Loukas A, Perry CL. Who is JUULing and why? An examination of young adult electronic nicotine delivery systems users. J Adolesc Health. 2020;66(1):48–55.
23. Meng YY, Ponce NA. The changing landscape: tobacco and marijuana use among young adults in California. [Internet]. UCLA Center for Health Policy Research, 2020. Available from: https://www.drugsandalcohol.ie/32302/.
24. Stone MD, Braymiller JL, Strong DR, Cwalina SN, Dimofte CV, Barrington-Trimis JL. Differentiating reasons for young adult e-cigarette use using maximum difference choice models. Nicotine Tob Res. 2023;25:1116–24.
25. Chok L, Cros J, Lebon L, Zürcher K, Dubuis A, Berthouzoz C, et al. Enquête sur l'usage et les représentations des cigarettes électroniques jetables (puffs) parmi les jeunes romand.es [Internet]. Centre universitaire de médecine générale et santé publique (Unisanté); 2023. Available from: https://serval.unil.ch/notice/serval:BIB_EF791F3BB6B3.
26. Jongenelis MI, Brennan E, Slevin T, Kameron C, Rudaizky D, Pettigrew S. Differences in use of electronic nicotine delivery systems by smoking status and demographic characteristics among Australian young adults. Health Promot J Austr. 2019;30:207–11.
27. Pettigrew S, Miller M, Alvin Santos J, Raj TS, Brown K, Jones A. E-cigarette attitudes and use in a sample of Australians aged 15–30 years. Aust N Z J Public Health. 2023;47(2): 100035.
28. Pettigrew S, Santos JA, Li Y, Miller M, Anderson C, Raj TS, et al. E-cigarette-related beliefs, behaviors, and policy support among young people in China. Tob Induc Dis. 2023;21: 09.
29. Newcombe KV, Dobbs PD, Oehlers JS, Dunlap CM, Cheney MK. College students' reasons for using JUULs. Am J Health Promot. 2021;35:835–40.
30. Freeman B, Owen K, Rickards S, Brooks A, Clare PJ, Dessaix A. E-cigarette use by people who smoke or have recently quit, New South Wales, 2016–2020. Med J Aust. 2023;218: 131–7.
31. Cooper M, Harrell MB, Perry CL. Comparing young adults to older adults in e-cigarette perceptions and motivations for use: implications for health communication. Health Educ Res. 2016;31:429–38.
32. Dunlop S, Lyons C, Dessaix A, Currow D. How are tobacco smokers using e-cigarettes? Patterns of use, reasons for use and places of purchase in New South Wales. Med J Aust. 2016;204:355.
33. Bunch K, Fu M, Ballbè M, Matilla-Santader N, Lidón-Moyano C, Martin-Sanchez JC, et al. Motivation and main flavour of use, use with nicotine and dual use of electronic cigarettes in Barcelona, Spain: a cross-sectional study. BMJ Open. 2018;8(3):e018329.

34. Khouja JN, Taylor AE, Munafò MR. Associations between reasons for vaping and current vaping and smoking status: Evidence from a UK based cohort. Drug Alcohol Depend. 2020; 217:108362.
35. Rostron BL, Yu-Ching Cheng, Gardner LD, Ambrose BK. Prevalence and reasons for use of flavored cigars and ENDS among US Youth and adults: estimates from wave 4 of the PATH Study, 2016-2017. Am J Health Behav. 2020;44(1):76–81.
36. Buu A, Hu YH, Wong SW, Lin HC. Comparing American college and noncollege young adults on e-cigarette use patterns including polysubstance use and reasons for using e-cigarettes. J Am Coll Health. 2020;68:610–6.
37. Patel D, Davis KC, Cox S, Bradfield B, King BA, Shafer P, et al. Reasons for current *E*-cigarette use among U.S. adults. Prev Med. 2016;93:14–20.
